# AI in Psychiatry: Patients Still See a Future for Human Psychiatrists

**DOI:** 10.1192/j.eurpsy.2026.10412

**Published:** 2026-07-31

**Authors:** G. Arbanas, M. Badurina, J. Sušac, A. Periša, I. Biliškov, D. Arbanas

**Affiliations:** 1 University Psychiatric Hospital Vrapče, Zagreb, Croatia; 2 BHIDAPA, Sarajevo, Bosnia and Herzegovina; 3 Codeasy, Split; 4 Karlovačke ljekarne, Karlovac, Croatia

## Abstract

**Introduction:**

Artificial intelligence (AI) has been used in psychiatry to generate medical content, to provide detailed drug-related information, to be a patient educatior, to generate summaries to identify diagnoses, list differential diagnoses and assessment procedures and provide holistic management plan. Research has shown that psychiatrists and professionals have optimistic views of the use of AI in psychiatry. Furthermore, lay subjects were willing to use ChatGPT for health advice.

**Objectives:**

This study aims at determining what patients with different mental disorders think about the use of chatbots in psychiatry, and about the advantages and problems of using them for mental health issues.

**Methods:**

Outpatients from two mental health institutions (one in Croatia and another in Bosnia and Herzegovina) were asked to assess the extent to which chatbots were perceived as professional, knowledgeable and easy to approch compared with human providers. Participants included 121 patients, 61% women, one third with neurotic, stress-related, and somatoform disorders, one third with schizophrenia-related disorders, and 20% with mood/affective disorders.

**Results:**

Participants reported the highest mean satisfaction with psychiatrists (4.67 out of 5), followed by pharmacists (3.94) and ChatGPT (3.66). There were no significant differences between the sexes. Patients noted availability and instantaneous answers as the main advantage of chatbots. The main disadvantage was the lack of personal contact (“it is not a real human being, has no emotions, and cannot provide contact”).

**Conclusions:**

Although chatbots can give precise and accurate responses and follow professional guidelines, patients need something more - support, empathy, and an actual relationship.

**Disclosure of Interest:**

None Declared

